# Pneumococcal Epidural Abscess in an Asplenic Patient Presenting After Mild Hip Trauma

**DOI:** 10.7759/cureus.7494

**Published:** 2020-03-31

**Authors:** Ronan Allencherril, Linda Joseph

**Affiliations:** 1 Internal Medicine, The University of Texas Medical Branch, Galveston, USA; 2 Anesthesiology, Baylor College of Medicine, Houston, USA

**Keywords:** spinal epidural abscess, asplenia, trauma, paraparesis

## Abstract

We report a unique presentation of an epidural abscess following mild trauma in a patient with asplenia. The patient reported subjective fever and marked pain along the right hip and flank, which are atypical locations for epidural abscess pain. A subsequent urinalysis showed leukocytes, and the diagnosis of an epidural abscess was missed until the patient presented over two weeks later with fever, spinal pain, leg weakness, and urinary incontinence. This report highlights the importance of heightened clinical suspicion of pneumococcal infections in asplenic patients with unexplained subjective fever. Cost-effective yet sensitive tests such as erythrocyte sedimentation rate (ESR) and C-reactive protein (CRP) can help guide further investigation of epidural abscesses in such patients. Blood and urine cultures may also be warranted. Early diagnosis of epidural abscesses is essential to ensure improved outcomes.

## Introduction

*Staphylococcus aureus *is responsible for approximately two-thirds of spinal epidural abscesses (SEA) incidence in the general population, while only about 1.5% is due to *Streptococcus pneumoniae* [1,2]. In patients with asplenia, disseminated pneumococcal infection is a relatively rare, but well-known complication [3]. Invasive pneumococcal infection often results in meningitis or pneumonia, and epidural abscesses are quite uncommon [4]. This case documents a unique presentation of pneumococcal SEA in the setting of urinary tract infection (UTI) and minor trauma in a patient with asplenia.

## Case presentation

**Figure 1:**
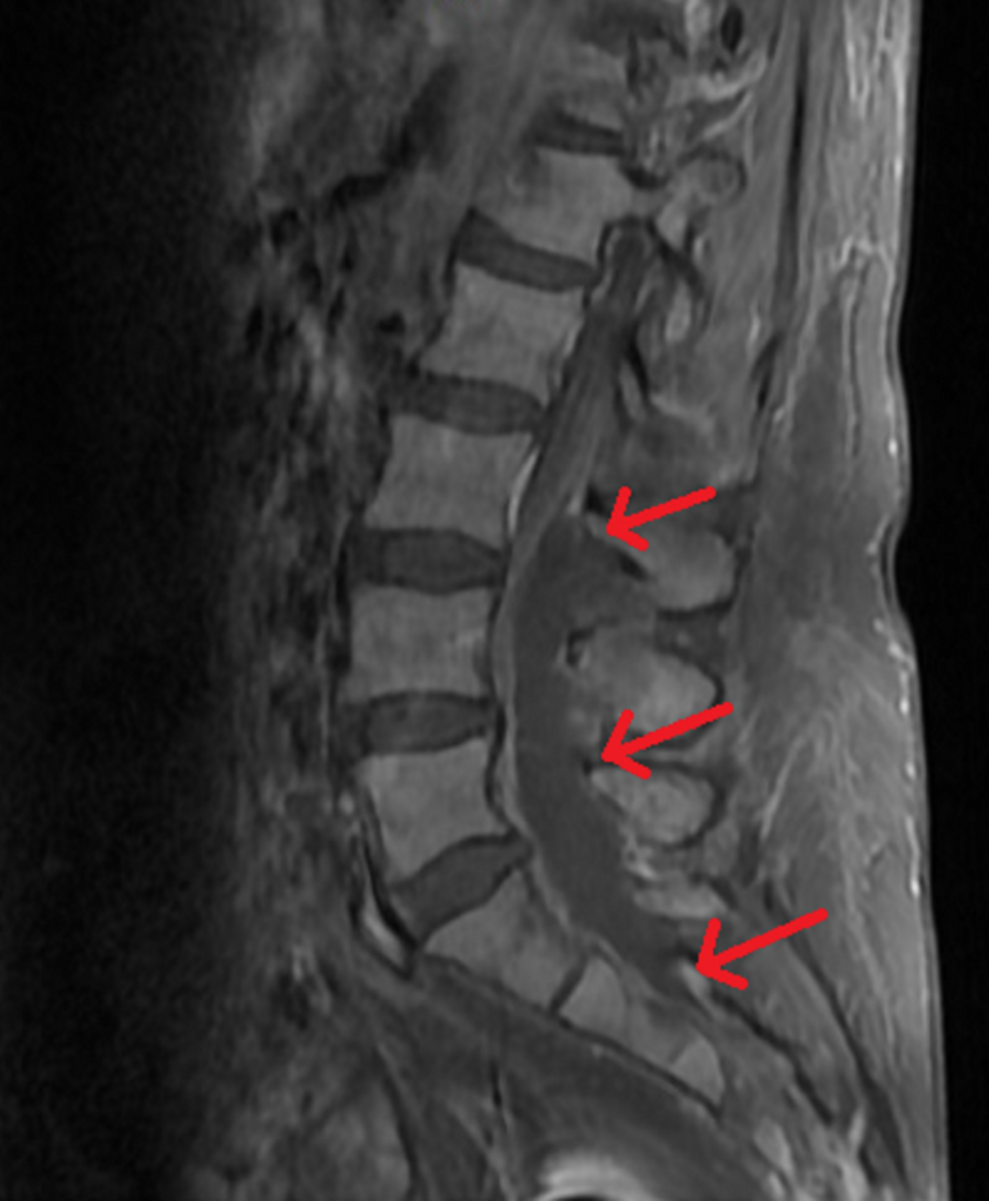
Lumbar MRI of the patient The image shows an extensive 10-cm epidural abscess from L3 to S2. Red arrows indicate the margins of the abscess MRI: magnetic resonance imaging

A 65-year-old Hispanic female with a past medical history of arthritis, hypertension (HTN), and splenectomy for refractory autoimmune hemolysis presented to the clinic complaining of right hip pain after accidentally hitting herself with the buckle of a seatbelt. The pain was immediate and described as severe and dull in character with radiation to the right flank. Tramadol provided moderate pain relief. On review of systems, she also admitted to occasional nausea, vomiting, and subjective fevers. She denied dysuria, hematuria, and urinary urgency. The patient had undergone combined cholecystectomy and splenectomy for refractory autoimmune hemolysis and subsequent development of gallstones and cholecystitis seven years ago. The patient’s autoimmune hemolysis had since been stable, and she was up-to-date with her vaccinations. Her HTN was well controlled with metoprolol tartrate. She had no history of intravenous (IV) drug use or back surgery/interventions. At the time of the visit, the patient was afebrile with unremarkable vitals. On physical exam, the right hip was tender to palpation with no signs of bruising or swelling. The range of motion of the back and hip was within normal limits. There was no paraspinal or costovertebral angle tenderness or suprapubic pain to palpation. Urinalysis was positive for leukocytes, and a presumptive diagnosis of UTI was made. A lumbar X-ray was ordered and the patient was sent home on ciprofloxacin. The X-ray showed no abnormalities at the one-week follow-up. At that time, the patient also noted moderate pain improvement compared to the first visit. Eight days after the follow-up, the patient presented to the emergency department (ED) for lower extremity weakness, left greater than the right, with associated urinary incontinence, vomiting, and loss of appetite. Her temperature was 39.3 ºC, and her pulse was 109; the rest of the vitals were within normal limits. Physical exam showed severe low-back pain, bilateral leg pain, and bilateral paraparesis with near-complete paralysis of the left leg. White blood cell (WBC) count was elevated at 25,300 cells/mm 3, with elevations in neutrophils, lymphocytes, and monocytes. Erythrocyte sedimentation rate (ESR) and C-reactive protein (CRP) were elevated at 114 mm/hr and 17.80 mg/L, respectively. A lumbar MRI showed an L3-S2 epidural abscess (Figure 1). 

**Figure 2:**
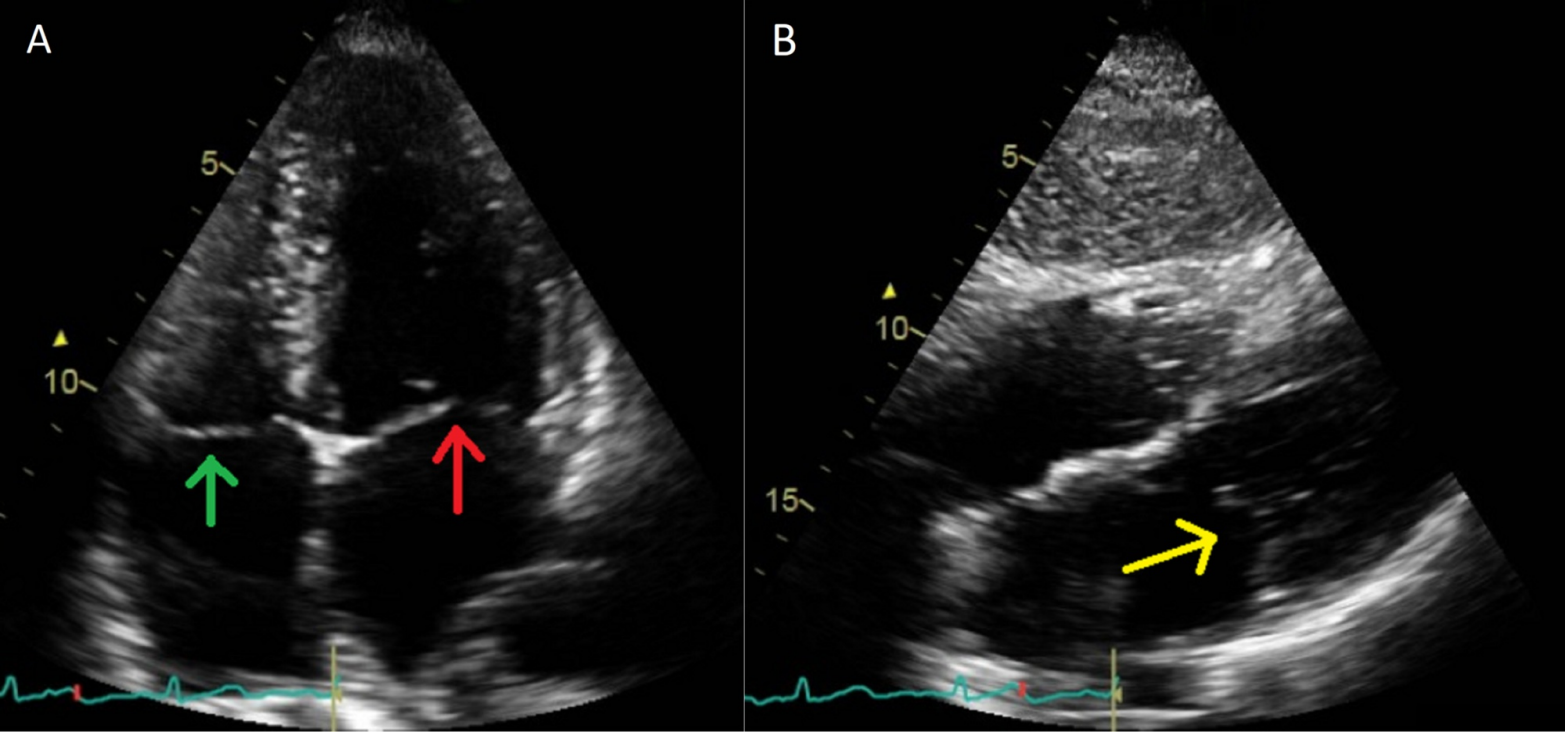
TTE images of the patient A: four chamber TTE; B; aortic longitudinal TTE. The images show no vegetations or abscesses involving any of the valves Green arrow: tricuspid valve; red arrow: mitral valve; yellow arrow: aortic valve TTE: transthoracic echocardiogram

**Figure 3:**
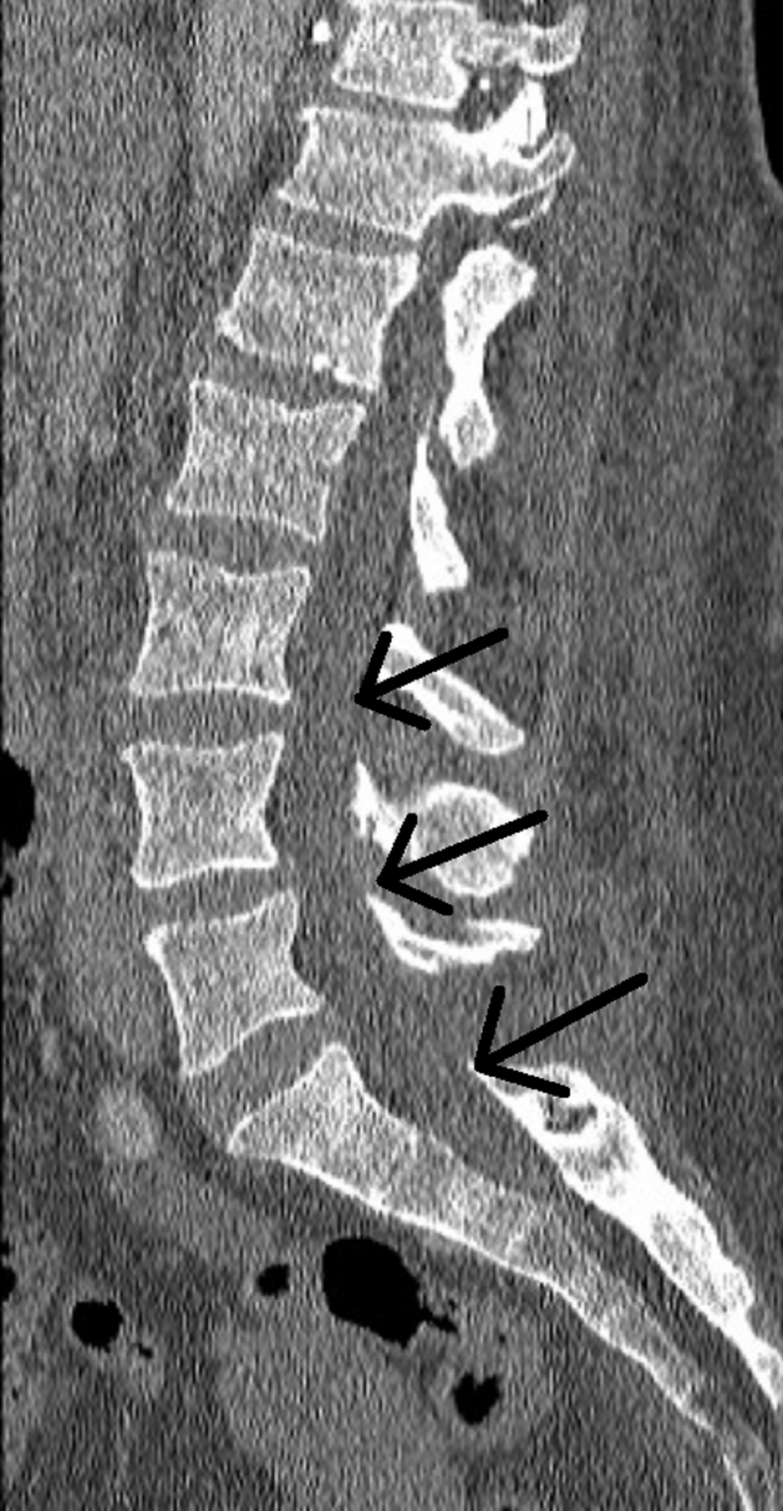
Lumbar CT with contrast at 16-day follow-up The image shows complete resolution of the epidural abscess and operative changes consistent with an L5/S1 laminectomy Black arrows indicate the former margins of the epidural abscess, now resolved CT: computed tomography

The patient was started on IV vancomycin and piperacillin-tazobactam. Neurosurgery was consulted and the patient underwent L5-S1 partial laminectomy with drainage of a purulent epidural abscess without complication. She was moderately delirious after the operation, and blood and abscess cultures came back positive for *Streptococcus pneumoniae*. Pathology showed granulation tissue and fibrinopurulent debris with no signs of malignancy. There was a concern for possible meningitis, so piperacillin-tazobactam was replaced with ceftriaxone. No lumbar puncture was performed due to the location of the abscess. A transthoracic echocardiogram (TTE) revealed mild concentric left ventricular hypertrophy and trace pulmonic and tricuspid insufficiency but no vegetations or abscesses (Figure 2). A chest X-ray showed mild pulmonary congestion and no signs of pneumonia. A follow-up CT lumbar showed resolution of the epidural abscess (Figure 3). The patient’s neurological deficits improved after surgery and rehabilitation. She received eight weeks of IV antibiotics and was discharged following rehab. The patient recovered at home uneventfully and passed away one year later from natural causes.

## Discussion

This case documents a unique presentation of a pneumococcal epidural abscess in an asplenic patient. Despite the history of subjective fevers, the patient’s initial clinic visit following minor trauma and a subsequent urinalysis suggestive of a UTI made it particularly difficult to suspect SEA.

Initial manifestations of SEA are classically associated with the triad of fever, spinal pain, and neurological deficits; however, in clinical practice, patients rarely present with all three symptoms simultaneously [5]. In our patient, this triad did not manifest until about two weeks after the initial visit, when she presented to the ED. Patients with this triad often indicate late presentation and poorer prognosis, and although this particular patient was discharged home without any long-term neurological complications, earlier diagnosis and treatment are imperative to improved outcomes [6]. The diagnosis was difficult in this case, as the patient only described nonspecific symptoms of undulating subjective fevers and right-sided hip and flank pain, rather than spinal pain. The positive urinalysis led the practitioner to believe that a UTI was the cause of the fever and flank pain. Although the patient did not have a history of osteoporosis, an X-ray was ordered to rule out the possibility of a traumatic fracture, but the film came back unremarkable. Therefore, in addition to the improvement in pain at follow-up, it was assumed that a simple UTI was the underlying issue and had successfully been cleared by the course of ciprofloxacin.

There are a few possibilities that could explain the patient’s course from presentation to surgery. In total, 18 days had passed between the patient’s initial clinic visit and the diagnosis of SEA by MRI. Strictly purulent drainage often suggests acute formation of the abscess over just a few days, whereas concomitant granulation tissue indicates a more prolonged course, likely at least two weeks [7,8]. Our patient’s pathology report showed both granulation tissue and fibrinopurulent debris suggestive of both chronic and acute inflammation. This, in addition to the patient’s subjective fever and flank and hip pain in the clinic, indicated a strong possibility that SEA had already formed when the patient first presented as an outpatient. This would allow for just over two weeks of abscess progression before neurological deficits appeared, which would not be unusual. It should be noted that the patient had admitted to an improvement of her symptoms at the follow-up visit in the week between the clinic visit and the ED visit, at which point she had been completing a course of ciprofloxacin for the UTI. Although *Streptococcus pneumoniae* is markedly less susceptible to ciprofloxacin relative to other fluoroquinolones, it is possible that ciprofloxacin’s poor potency had been enough to slow the progression of SEA and provide moderate improvement in the symptoms [9,10]. Approximately one week after completing the course of antibiotics, the patient presented to the ED with worsening spinal pain, fever, leg weakness, and incontinence. Overall, this picture indicates the presence of SEA at the initial clinic visit, although flank and hip would be atypical locations for the pain. However, the likelihood of minor trauma inflicting such severe pain is unlikely in a healthy patient, suggesting an underlying pathology such as SEA.

Another possibility, albeit less likely, was that the trauma from the seatbelt had resulted in the formation of SEA. Blunt trauma reportedly precedes SEA in 15-35% of cases, possibly due to epidural hematoma formation and subsequent infection [11-14]. Our patient first presented to the clinic after accidentally hitting herself on the right hip with the seatbelt. Still, it should be mentioned that in the ED, she described having hit herself in the back, rather than the hip, although the logistics of the former seem less plausible. In either case, the trauma had clearly lacked the degree of force necessary to cause a hematoma.

Although very unlikely, it is worth considering that the patient’s UTI had been caused by *Streptococcus pneumoniae* and, following inadequate treatment with ciprofloxacin, resulted in bacteremia and subsequent SEA. While there are reported cases of pneumococcal UTI in adults, they are extremely rare in immunocompetent patients [15,16]. This source of infection is only worth considering due to our patient’s asplenia. However, urine culture from the inpatient stay was positive only for yeast.

This patient may have benefited from further testing at the initial presentation. Given the patient's asplenia, there should be a greater clinical concern for more serious underlying infections. ESR and CRP can be used to rule out SEA due to the high sensitivity of the tests [17]. This would be more cost-effective than an MRI, which would only be pursued based on clinical suspicion and positive inflammatory markers.

## Conclusions

This case documents a unique presentation of SEA. At this patient’s initial visit, it had appeared that the subjective fevers and flank could be easily explained as caused by UTI, and further investigation was not conducted. The clinician did not consider epidural abscess on the list of differential diagnoses. Although the patient was later treated and recovered adequately without long-term sequelae, early diagnosis is always the standard of care for epidural abscesses in improving prognosis. This is not to suggest that physicians should pursue workups of red-herring diagnoses, but rather that patients with asplenia and unexplained fevers may warrant a lower threshold for further investigation for a more serious underlying infection. Although our patient had SEA, the same caution should be applied for abscesses elsewhere, bacteremia, or meningitis. While MRI is the gold standard for the diagnosis of SEA, more cost-effective tests such as ESR and CRP have been shown to be highly sensitive in ruling out SEA in patients with back pain. There should be close follow-ups with asplenic patients presenting with fever, especially if antibiotics are prescribed. In the case of our patient, perhaps a more detailed history and physical, in addition to ESR, CRP, hemogram, and even blood and urine cultures may have been warranted for an earlier diagnosis.
